# Depression Promotes Gastroesophageal Reflux Disease: New Evidence Based on Mendelian Randomization

**DOI:** 10.5152/tjg.2023.22231

**Published:** 2023-05-01

**Authors:** Gui Chen, Junyang Xie, Jinfeng Ye, Xiaoxuan Kuang, Wenjing Liao, Lijuan Song, Xiaowen Zhang

**Affiliations:** 1 Department of Otolaryngology-Head and Neck Surgery, State Key Laboratory of Respiratory Disease, the First Affiliated Hospital of Guangzhou Medical University, Guangzhou, Guangdong, China

**Keywords:** Depression, gastroesophageal reflux disease, Mendelian randomization, UK Biobank

## Abstract

**Background:**

Although observational studies have reported that depression is a risk factor for gastroesophageal reflux disease, it is difficult to determine the potential causal correlation. Thus, this study investigated the causal relevance of depression for gastroesophageal reflux disease using Mendelian randomization and provided new evidence for their association.

**Methods:**

Based on data from the UK Biobank, we assessed the causality of the 2 diseases by analyzing 135 458 severe depressive disorder cases and 41 024 gastroesophageal reflux disease cases. The causal inference was assessed using inverse-variance weighting, weighted median, Mendelian randomization–Egger, and weighted median methods. Simultaneously, pleiotropy and sensitivity analyses were used for quality control. Finally, we also explored whether depression affects gastroesophageal reflux disease through other risk factors.

**Results:**

A positive causal relationship between depression and gastroesophageal reflux disease was found in the inverse-variance weighted and weighted median methods, both of which were statistically significant [odds ratio = 1.011, 95% CI: 1.004-1.017, *P*  = .001; odds ratio = 1.011, 95% CI: 1.004-1.020, *P*  = .002)]. Sensitivity analyses were consistent with a causal interpretation, and the main deviation of genetic pleiotropy was not found (Intercept *β* = 0.0005; SE = 0.005, *P*  = .908). The genetic susceptibility to depression was also associated with smoking, insomnia, and sleep apnea (odds ratio = 1.166, 95% CI: 1.033-1.316, *P*  = .013; odds ratio = 1.089, 95% CI: 1.045-1.134; and odds ratio = 1.004, 95% CI: 1.001-1.006, *P*  = .001, respectively).

**Conclusion:**

Our results verified a causal correlation that depression could slightly increase the risk of gastroesophageal reflux disease.

**Main Points**

- Major depressive disorder (MDD) and gastroesophageal reflux disease (GORD) are common diseases, and there is a close relationship between the 2 diseases.
- The results of observational studies on MDD and GORD are inconsistent.
- Previous evidence of MDD and GORD causality are from observational studies.
- Our results verified a causal correlation that depression could slightly increase the risk of GORD.

## Introduction

Gastroesophageal reflux disease (GORD) is a common disease caused by the reflux of gastric contents into the esophagus, causing discomfort symptoms and/or complications inside and outside the esophagus.^1,2^ Several observational studies have reported that genetic predisposition, smoking, drinking, insomnia, sleep apnea, hypertension, type 2 diabetes, obesity, and psychological abnormalities are common risk factors for developing GORD.^1,3-6^ Major depressive disorder (MDD) is a common psychological disease characterized by depression, decreased interest, and impaired cognitive function.^7^ Both the incidence and prevalence of depression and GORD have been increasing, eventually becoming a global health concern in recent years.^8,9^ Previous studies have found that depression and GORD are closely related and that there is an interaction between the 2 diseases.^10-13^ Systematic reviews have shown that the severity and frequency of GORD symptoms are closely related to the severity of depression.^14^ However, there are also unanswered key questions in depression and GORD, particularly regarding potential mechanisms underlying this comorbidity. Currently, it is unclear whether the association between GORD and depression comes from common genetic or environmental factors and whether these associations are causal.

Mendelian randomization (MR) with genetic association is a new epidemiological tool for assessing causality that cannot be confused with other risk factors.^15^ This method can provide strong causal evidence for the research and can effectively avoid the deviation caused by observational research.^16^ An MR study has been used to evaluate the effect of higher central adiposity on GORD, indicating that a higher waist–hip ratio increases the risk of GORD.^17^ In this study, we evaluated the potential causal role of depression in GORD and explored the potential mediators of the causal association between the two.

## Materials and Methods

### Genetic Variants Associated with Major Depressive Disorder

A meta-analysis of MDD in the genome-wide association studies (GWAS) was applied as an exposure set in this study, with 135 458 MDD cases and 344 901 controls among participants.^18^ The MDD cases were diagnosed using the Diagnostic and Statistical Manual of Mental Disorders-IV or the International Classification of Diseases-10 (ICD-10). Based on strict inclusion criteria (*P*  < 5 × 10^−8^), Wray et al^18^ reported that 44 single nucleotide polymorphisms (SNPs) played a significant role in MDD (Table S1). The 0.23% change in MDD was explained by these associated SNPs, and the instruments with large F‐statistics (value of 156) can strongly predict GORD.^19^

### Genome-Wide Association Study Summary Data on Gastroesophageal Reflux Disease

Gastroesophageal reflux disease’s genome-wide association study summary data were from the UK Biobank (https://www.ukbiobank.ac.uk/).^20^ The GORD cases were defined by medical record ICD10. Totally, there were 41 024 GORD cases and 410 073 controls. There were 15 SNPs removed from the 44 SNPs in MDD, including palindromic with intermediate allele frequency and the result set lacked.

### Summary Data of Gastroesophageal Reflux Disease Risk Factors

The association of MDD with previously reported risk factors for GORD was assessed by inverse variance weighting (IVW) to explore whether depression affects GORD through other risk factors. Smoking, drinking, body mass index, daytime sleeping, sleeplessness/insomnia, sleep apnea, hypertension, and type 2 diabetes were considered as possible mediating factors of GORD. The data of these factors were obtained from several widely used databases (Table 1).

### Statistical Analyses

According to MR guidelines, the IVW method has maximum statistical power when the instrumental variables (IVs) are all effective. Therefore, it was mainly used to evaluate the causal relationship between MDD and GORD risk.^21^ If at least 50% of the weights in the analysis is from IVs, the weighted median method provides a causal estimation.^22^ Even if all SNPs are ineffective tools, the MR–Egger method provides an effective estimation^23^. In addition, the MR–Egger Regression was used to evaluate horizontal pleiotropy. The robustness of significant results was evaluated by the leave-one-out sensitivity test. All MR analyses in this study were performed using R (version 4.0.3) with the “TwoSampleMR” package (R Foundation for Statistical Computing, Vienna, Austria).^24^

## Results

**Figure 1.**
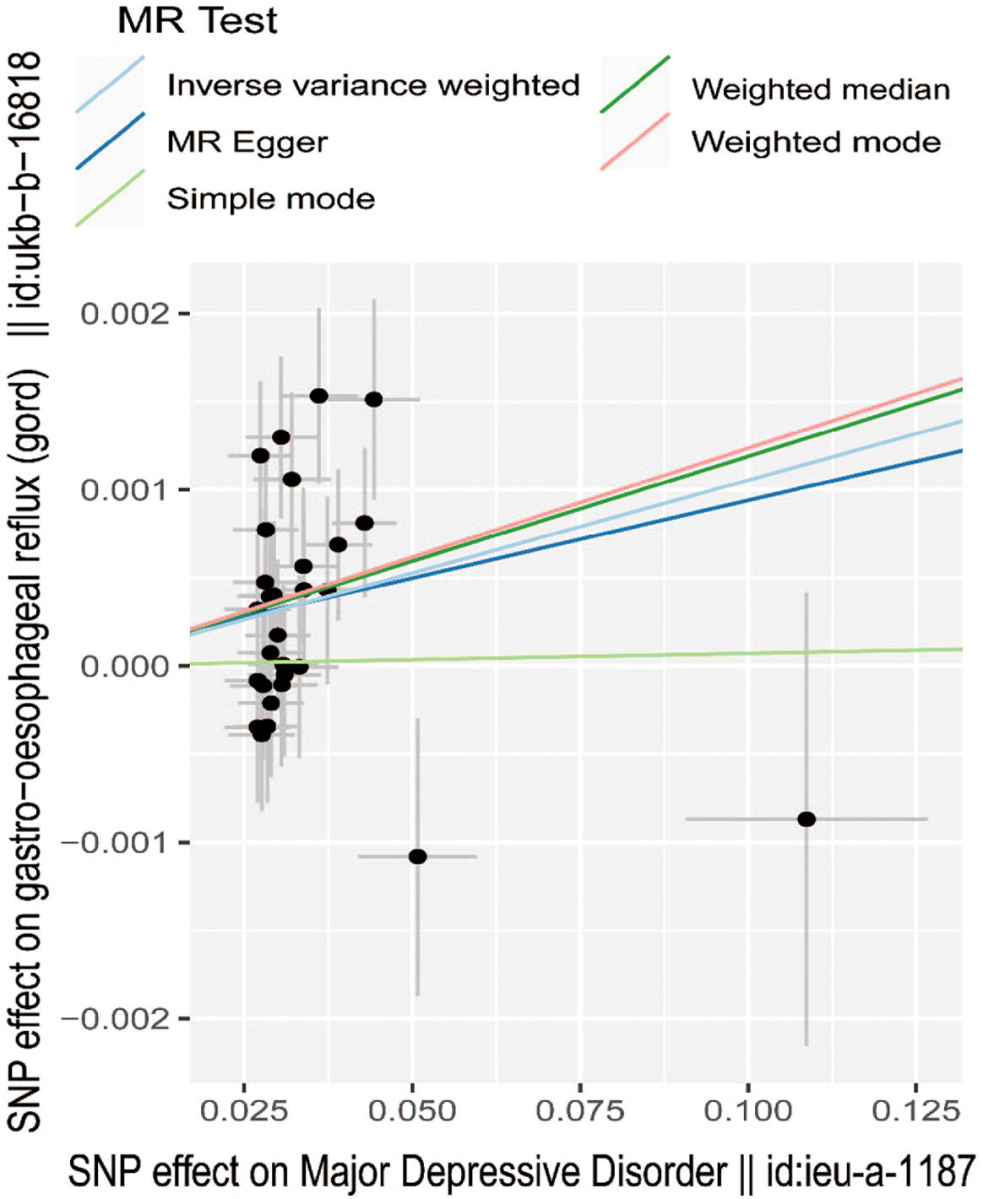
Scatter plot of the effect of SNPs on major depressive disorder and gastro-oesophageal reflux disease. SNP, single-nucleotide polymorphism; MR, Mendelian randomization.

A total of 29 SNPs associated with MDD were used. Patients with MDD were 1.011-fold more likely to have GORD (95% CI: 1.004-1.017, *P*  = .001) than those without MDD. In addition, a similar positive result was also found in the weighted median method (odds ratio [OR] = 1.011, 95% CI: 1.004-1.020, *P*  = .002) (Figure 1). At the same time, the MR–Egger method and weighted mode obtained similar risk estimates, but neither was found to be statistically significant.

**Figure 2.**
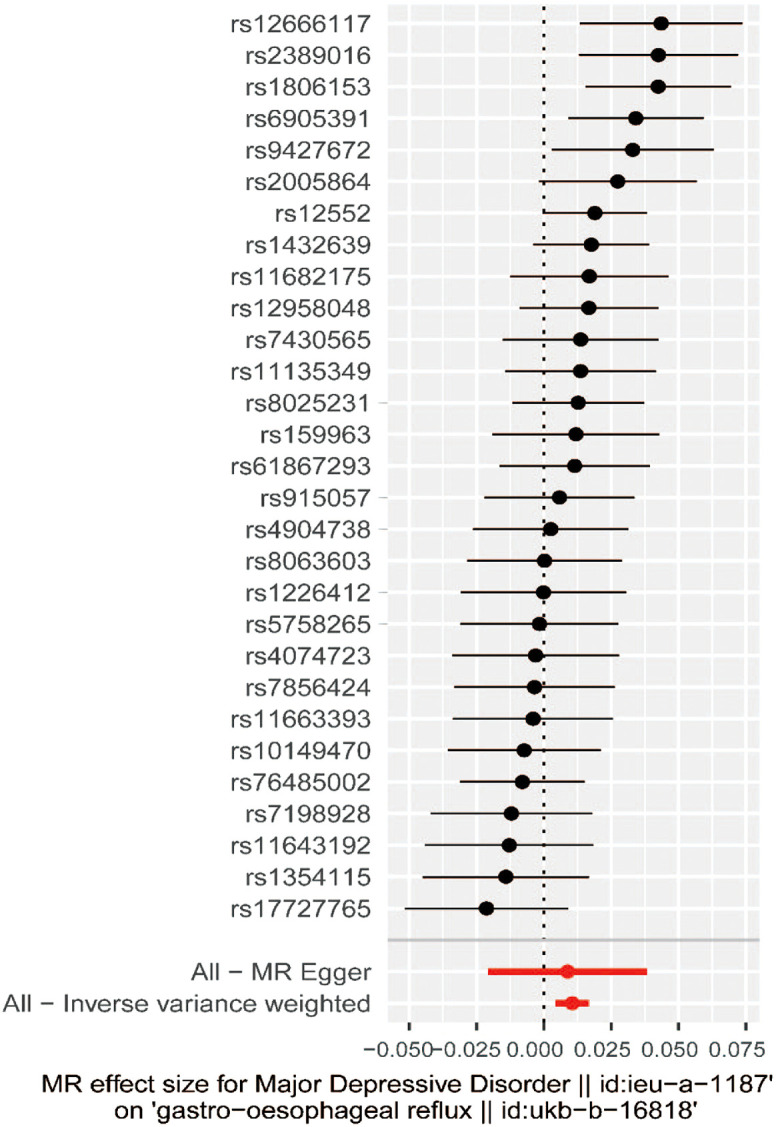
Forest plot of SNPs associated with major depressive disorder and their risk of gastro-oesophageal reflux. SNP, single-nucleotide polymorphism; MR, Mendelian randomization.

**Figure 3.**
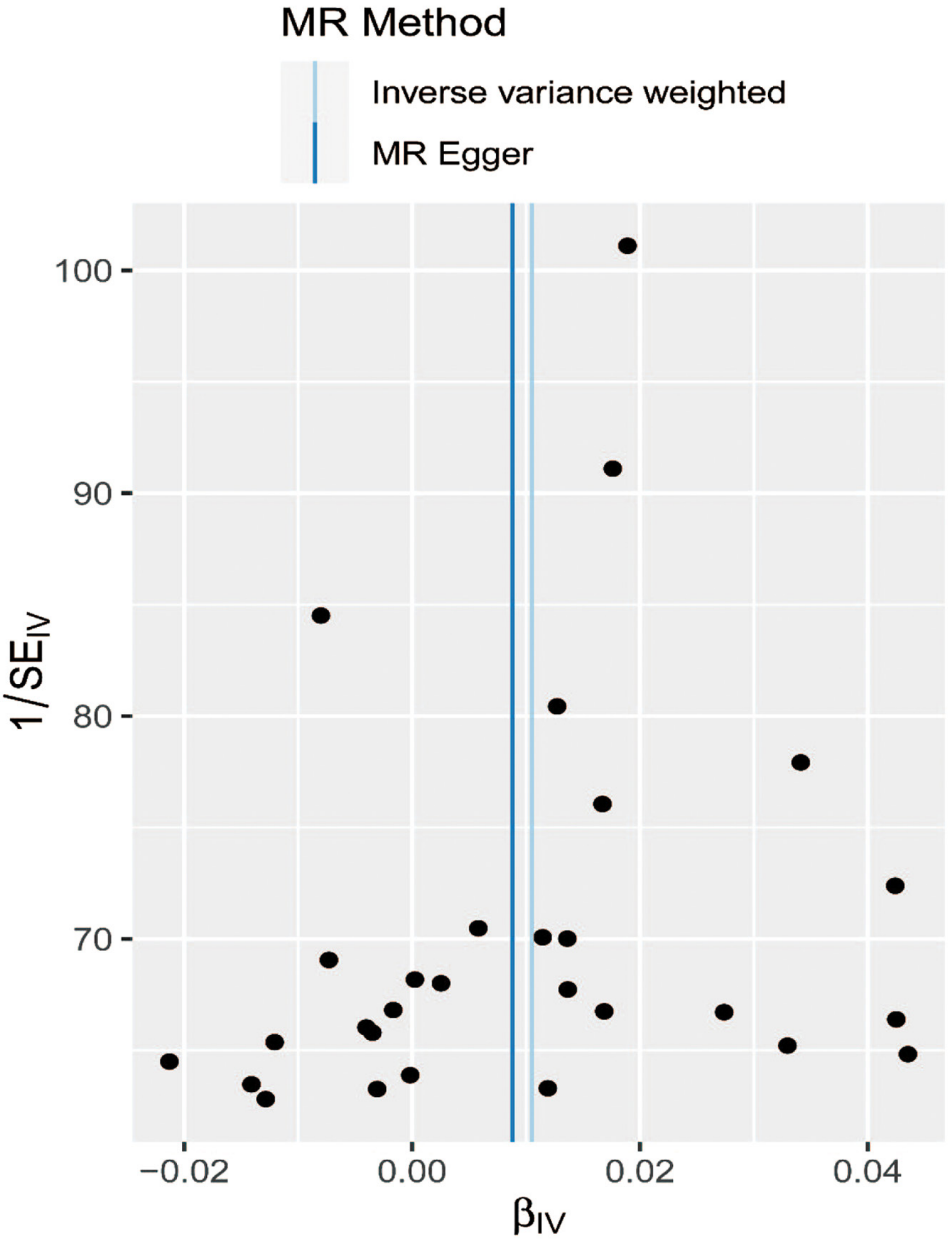
Funnel plot of SNPs associated with major depressive disorder and their risk of gastro-oesophageal reflux disease. SNP, single-nucleotide polymorphism; MR, Mendelian randomization; IV, instrumental variable; 1/SE_IV_, instrument precision; β_IV_, in (OR) estimates.

**Figure 4.**
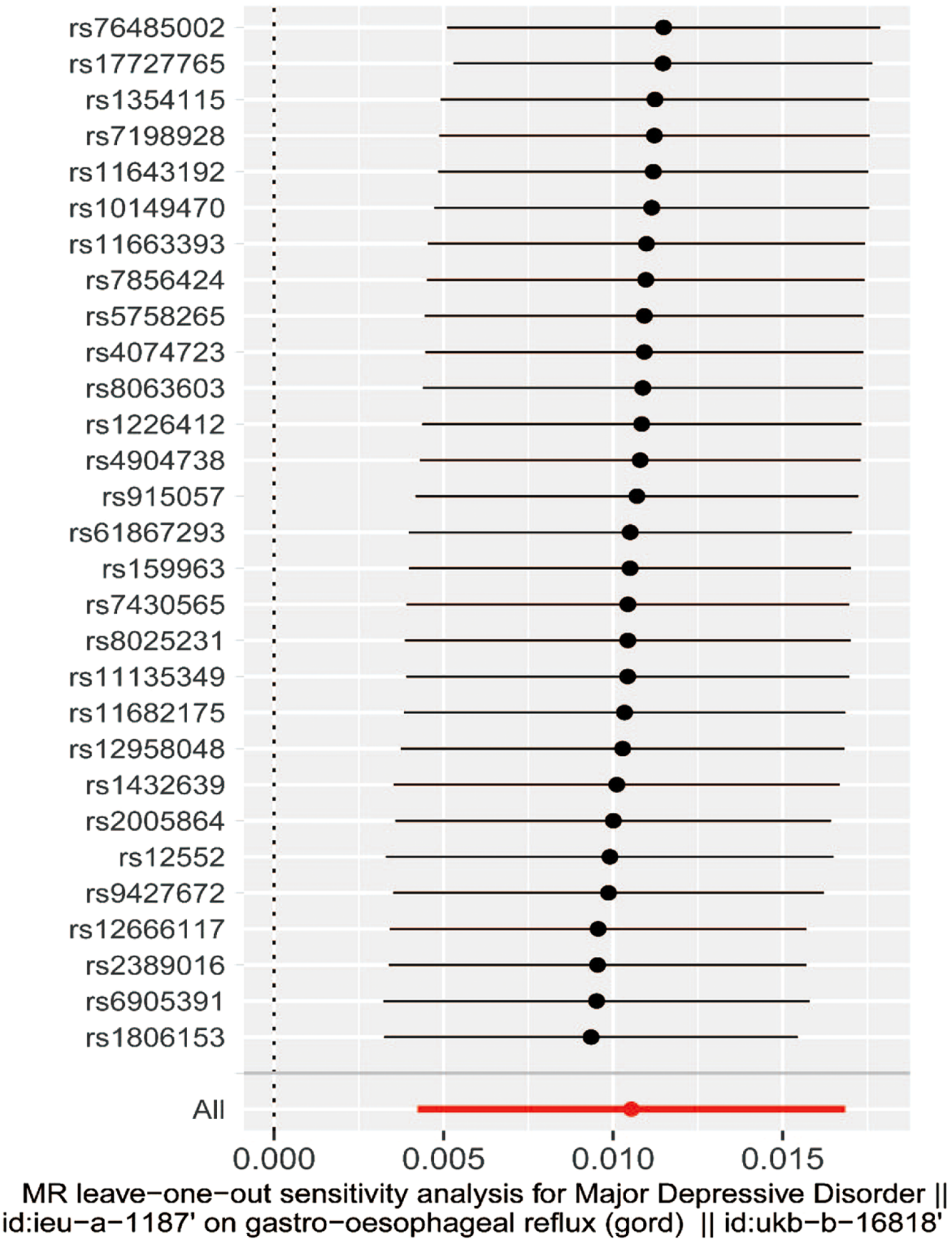
Leave-one-out sensitivity analysis of SNPs associated with major depressive disorder and risk of gastro-oesophageal reflux disease. SNP, single-nucleotide polymorphism; MR, Mendelian randomization.

The MR–Egger method is usually used to test whether multiple IVs have horizontal pleiotropy. No significant intercept was found in this study (intercept *β* = 0.0005; SE = 0.005, *P*  = .908), indicating the lack of horizontal pleiotropy. (Figure 2). The funnel plot can intuitively show the heterogeneity of SNPs; the funnel plot in this study was symmetrical as a whole, showing that there was no pleiotropy (Figure 3). The omission sensitivity analysis showed that the MR results were actually robust and not affected by the SNP (Figure 4).

Furthermore, the IVW also assessed the relationship between depression and previously reported risk factors for GORD to explore the potential factors that serve as mediators (Table 2). A statistical significance was found in smoking, sleeplessness/insomnia, and sleep apnea (OR = 1.166, 95% CI: 1.033-1.316, *P*  = .013; OR = 1.089, 95% CI: 1.045-1.134, *P*  < .001; and OR = 1.004, 95% CI: 1.001-1.006, *P*  = .001, respectively).

## Discussion

In this study, the causal relationship between depression and GORD was verified using publicly available summary statistics from several genetic consortia. The MR analysis showed that depression could slightly promote GORD. This result was conducive to a clear understanding of the relationship between depression and GORD, which may contribute to better prevention and treatment of GORD.

Observational studies have shown that depression can lead to an increased risk of reflux symptoms.^25,26^ Studies have shown that mental disorders are associated with the onset of reflux esophagitis, but no correlation was found between the severity of depression and the severity of reflux esophagitis.^27^ In addition, a study showed that patients with anxiety and depression had a 2.8-fold higher risk of GORD than those without anxiety and depression.^25^ In one study, patients with GORD with underlying mental disorders had postoperative satisfaction of 11.1%, compared with the 95.3% in patients without these diseases.^28^ Therefore, depression is usually considered to be a potential factor of GORD in observational studies. Unfortunately, due to the inevitable limitations of observational studies, the causal relationship between these diseases is difficult to be confirmed. Based on this background, MR analysis may be an important causal reasoning research tool. Through MR analysis, our results supported the hypothesis that depression is a risk factor for GORD.

It should be noted that the pathophysiological mechanism of the relationship between depression and GORD remains unclear. Existing studies point to the following possible explanations. Due to the influence of psychological factors, patients with depression can reduce the sensory threshold of the body and increase the sensation of esophageal stimulation.^29,30^ Studies have also shown that there is a close relationship between the brain and the gastrointestinal tract.^31^ Patients with depression may have increased lower esophageal sphincter relaxation and then aggravated GORD.^32^ The mechanism of psychological factors affecting reflux symptoms has also been confirmed in animal studies. The damage to esophageal epithelial tight junction in stressed rats leads to the decreased functionality of the esophageal mucosal barrier, which then increases the vulnerability of the esophagus to reflux.^33^ Some antidepressants may reduce the pressure of lower esophageal sphincter, cause esophageal dysfunction, and aggravate reflux symptoms.^34,35^ Patients with depression may alleviate depressive symptoms through an unhealthy lifestyle, and factors such as smoking, drinking, and unhealthy eating habits may increase the risk of GORD in patients with depression.^32,36^

We further explored whether some intermediate phenotypes play a role in the causal relationship between depression and GORD. We focused on some intermediate phenotypes and found that smoking, insomnia, and sleep apnea may play a role. In addition, a previous MR study showed that higher central adiposity is the causal determinant of the risk of GORD.^17^

Our study had several limitations. First, the related GWAS data were from populations of European ancestry, and GORD-related individuals were middle-aged and elderly people in the UK. Second, more result datasets related to GORD in MR analysis had not been further verified. Third, there were multiple subtypes in patients with depression and GORD, and further subgroup analysis may be necessary.

## Conclusions

This study demonstrated that there is a causal relationship between depression and GORD and that depression could slightly increase the risk of GORD.

## Figures and Tables

**Table 1.** Details of the Risk Factors Related to GWASs Included in Mendelian Randomization

Phenotype	Consortium	Participants	PMID/Web Source
Major depressive disorder	A meta‐analysis of GWAS	480 359	29700475
Body mass index	A meta‐analysis of GWAS	681 275	30124842
Daytime dozing/sleeping (narcolepsy)	Neale Lab Consortium	336 082	http://www.nealelab.is/uk‐biobank
Hypertension	Neale Lab Consortium	361 194	http://www.nealelab.is/uk‐biobank
Type 2 diabetes	A meta‐analysis of GWAS	701 27	29358691
Cigarettes per day	GWAS and Sequencing Consortium of Alcohol and Nicotine use	337 334	30643251
Alcoholic drinks per week	GWAS and Sequencing Consortium of Alcohol and Nicotine use	335 394	30643251
Sleeplessness/insomnia	Neale Lab Consortium	336 965	http://www.nealelab.is/uk‐biobank
Sleep apnoea	Neale Lab Consortium	361 194	http://www.nealelab.is/uk‐biobank

BMI, body mass index; GWAS, genome-wide association studies.

**Table 2.** Relationship Between Major Depressive Disorder Predicted by Genetic Data and Potential Risk Factors of GORD

Outcomes	nSNPs	OR	95% CI	*P*
Body mass index	27	1.022	0.917-1.140	.689
Daytime dozing/sleeping (narcolepsy)	32	1.019	0.999-1.040	.068
Hypertension	32	1.000	0.998-1.002	.884
Type 2 diabetes	32	1.031	0.854-1.244	.753
Cigarettes per day	31	1.166	1.033-1.316	.013
Alcoholic drinks per week	31	1.000	0.955-1.048	.992
Sleeplessness/insomnia	32	1.089	1.045-1.134	<.001
Sleep apnoea	36	1.004	1.001-1.006	.001

nSNP, number of single-nucleotide polymorphism; OR, odds ratio; GORD, gastroesophageal reflux disease.

**Supplementary Table 1.** Forty-four SNPs Associated with Major Depressive Disorder

SNP	Chromosome	Position	Effect allele	Other allele	Frequency	Beta	Se	P	Sample size
rs1432639	1	72813218	A	C	0.6187	-0.0027	0.0083	.746	140, 254
rs159963	1	8504421	A	C	0.5671	0.0051	0.0085	.5495	140, 254
rs2389016	1	80799329	T	C	0.2744	4.00E-04	0.0096	.9632	140, 254
rs4261101	1	90796053	A	G	0.3543	-0.0036	0.0086	.6699	140, 254
rs9427672	1	1.98E+08	A	G	0.2187	-0.018	0.0101	.07444	140, 254
rs11682175	2	57987593	T	C	0.5333	-0.0178	0.008	.02626	140, 254
rs1226412	2	1.57E+08	T	C	0.7859	-0.005	0.01	.6207	140, 254
rs9862324	3	44433910	T	C	0.669	-0.0085	0.0086	.326	140, 254
rs7430565	3	1.58E+08	A	G	0.5674	0.0027	0.008	.7403	140, 254
rs34215985	4	42047778	C	G	0.2135	-0.0179	0.01	.07425	140, 254
rs27732	5	87992576	A	G	0.4167	-0.0081	0.0083	.3302	140, 254
rs2018142	5	1.04E+08	A	C	0.5229	-0.0085	0.0081	.2922	140, 254
rs116755193	5	1.24E+08	T	C	0.3786	-0.0193	0.0085	.02314	140, 254
rs11135349	5	1.65E+08	A	C	0.4504	0.0207	0.0083	.01254	140, 254
rs4869056	5	1.67E+08	A	G	0.6311	-5.00E-04	0.0089	.9554	140, 254
rs115507122	6	30737591	C	G	0.172	-0.0526	0.0106	.0000	140, 254
rs9402472	6	99566521	A	G	0.2222	-0.006	0.0099	.5395	140, 254
rs10950398	7	12264871	A	G	0.4074	0.011	0.0081	.1762	140, 254
rs12666117	7	1.09E+08	A	G	0.4652	-0.0034	0.008	.6718	140, 254
rs1354115	9	2983774	A	C	0.6308	0.0092	0.0089	.3049	140, 254
rs10959913	9	11544964	T	G	0.765	-0.0231	0.0098	.01801	140, 254
rs7856424	9	1.20E+08	T	C	0.2896	0.0087	0.0091	.3379	140, 254
rs7029033	9	1.27E+08	T	C	0.0796	-0.0189	0.0151	.2121	140, 254
rs61867293	10	1.07E+08	T	C	0.206	0.0127	0.0099	.2009	140, 254
rs1806153	11	31850105	T	G	0.225	-1.00E-04	0.0099	.9951	140, 254
rs4074723	12	23947737	A	C	0.401	-0.0168	0.0091	.06559	140, 254
rs4143229	13	44327799	A	C	0.9189	-0.0093	0.0148	.5284	140, 254
rs12552	13	53625781	A	G	0.4329	0.0051	0.0084	.5459	140, 254
rs4904738	14	42179732	T	C	0.555	-0.009	0.0082	.2716	140, 254
rs915057	14	64686207	A	G	0.4302	-0.0163	0.0083	.05126	140, 254
rs3742786	14	75373011	A	G	0.4488	0.0242	0.0079	.002278	140, 254
rs10149470	14	1.04E+08	A	G	0.4949	-0.0213	0.008	.007901	140, 254
rs8025231	15	37648402	A	C	0.5519	0.0041	0.0083	.6174	140, 254
rs8063603	16	6310645	A	G	0.6568	-0.0047	0.0089	.6002	140, 254
rs7198928	16	7666402	T	C	0.6256	-0.0049	0.0084	.5638	140, 254
rs7200826	16	13066833	T	C	0.2605	-0.0089	0.0096	.3528	140, 254
rs11643192	16	72214276	A	C	0.4027	-0.0234	0.0082	.004284	140, 254
rs17727765	17	27576962	T	C	0.9147	-0.012	0.015	.4247	140, 254
rs62099069	18	36883737	A	T	0.4246	-0.0097	0.0084	.2505	140, 254
rs11663393	18	50614732	A	G	0.4713	0.0079	0.008	.3242	140, 254
rs1833288	18	52517906	A	G	0.7116	0.0192	0.0095	.04256	140, 254
rs12958048	18	53101598	A	G	0.3223	-0.0151	0.0086	.07798	140, 254
rs5758265	22	41617897	A	G	0.2832	0.0173	0.0089	.05145	140, 254

SNP, single-nucleotide polymorphism.
